# Recent progress in antimicrobial strategies of controlled-release nanomaterials for secondary caries

**DOI:** 10.3389/fcimb.2025.1669643

**Published:** 2025-10-01

**Authors:** Yiyi Wang, Xushuo Du, Yanmin Jia, Lu Qin, Fei Liu, Yingchun Cai, Suping Wang

**Affiliations:** ^1^ Stomatology Center, The First Affiliated Hospital of Zhengzhou University, Zhengzhou, China; ^2^ School of Stomatology, Zhengzhou University, Zhengzhou, China; ^3^ Department of Stomatology, The First Affiliated Hospital of Zhengzhou University, Zhengzhou, China; ^4^ Department of Orthopedics, The First Affiliated Hospital of Zhengzhou University, Zhengzhou, China

**Keywords:** antimicrobial, nanomaterials, controlled-release, stimulus-responsive, rechargeable, cascade catalytic nanoreactor, secondary caries

## Abstract

Secondary caries is a leading cause of restoration failure. Inhibiting caries through antimicrobial efficacy is essential for extending the restoration’s service life. Antimicrobial agents have been incorporated into restorative materials for decades. Based on their mechanism of antimicrobial action, these materials are classified as either releasing or non-releasing types. However, the simple release strategy is often insufficient for long-term caries prevention, as it lacks the precision, durability, and adaptability now required. This necessitates the development of next-generation systems that can provide a controlled, sustained, and targeted antimicrobial activity. To this end, this review focuses on advanced, controlled-release antimicrobial strategies, exploring the design of novel nanomaterials, their functional efficacy, and the mechanisms of their representative antimicrobial agents.

## Graphical Abstract

These figures were created with BioGDP.com.

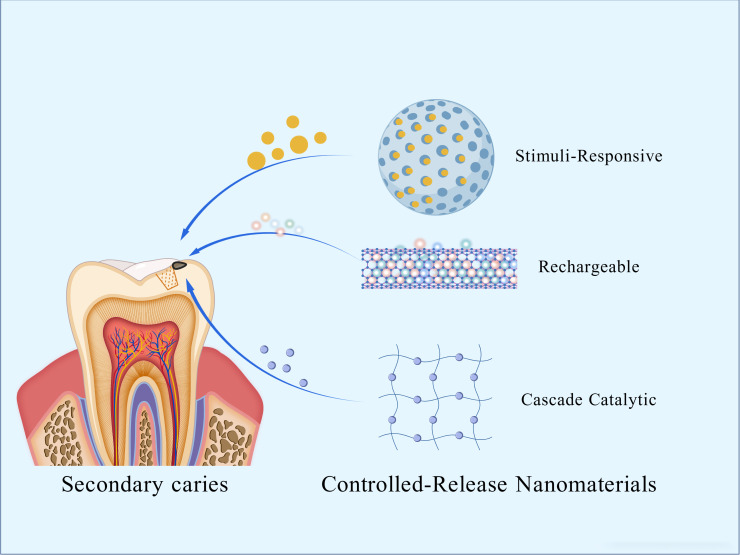

## 1 Introduction

Secondary caries is a common reason associated with failed restorations. Factors such as polymerization shrinkage of composite resin materials, mechanical stress, and adhesive degradation can lead to microgaps forming between the restoration and the tooth structure (Alizadeh Oskoee et al., 2017). The polymerization shrinkage of composite resins, combined with mechanical stress and adhesive degradation, can create micro-gaps at the restoration-tooth interface (Bowen et al., 2018). These marginal defects allow acid-producing bacteria to penetrate, metabolizing carbohydrates into a localized acidic microenvironment. Once established, this process of secondary caries accelerates the loss of tooth structure and elevates the risk that pulp treatment will be required (Schwendicke et al., 2021). While modern restorative materials can meet functional restoration needs, they cannot actively address bacterial infections at vulnerable margins or demineralization issues.

Early efforts to extend the service life of dental composites centered on enhancing their mechanical properties and minimizing polymerization shrinkage (Zhang et al., 2015; Balhaddad et al., 2021). Advances in nanotechnology have spurred interest in multifunctional composites, marking a pivotal shift from passive to active strategies in preventing secondary caries. Antimicrobial composites reduce bacterial adhesion via antimicrobial release or contact-killing, preventing biofilm formation (Jiao et al., 2019; Xue et al., 2020). Composites designed for remineralization can provide ion sources: calcium and phosphate to compensate for structural damage caused by demineralization in early-stage caries (Besinis et al., 2014). Traditional antimicrobial agents or remineralization ions are often released linearly or uncontrolled, resulting in short release durations and uncontrollable release quantities (Sivakumar et al., 2014). However, with the continuous development of nanotechnology, this issue has been effectively addressed, enabling precise control over the release of antimicrobial agents and remineralization ions (Rostami et al., 2025). By carefully designing the structure of nanomaterials to respond to specific stimuli, targeted release can be achieved (Wang et al., 2024). Rechargeable nanomaterials allow for the repeated release of bioactive agents, while sophisticated cascade nanoreactors enable a synergistic, multi-step process of antibacterial action and remineralization within the caries microenvironment. Crucially, nanoparticles preserve the mechanical strength of restorative materials while introducing new functionalities (Amin et al., 2025).

Therefore, this paper reviews the latest progress of controlled-release antimicrobial nanomaterials (as summarized in **Table 1**) in preventing and treating secondary caries from the perspective of the strategy mechanism.

**Table 1:** Controlled-release nanomaterials.

Type	Nanomaterial	Material type	Mechanism	Release kinetics	Advantages/ disadvantages	Author (year)
pH Responsive	BioUnion	Bioactive glass powder	Release of Zn^2+^, F^−^, Ca^2+^	Significantly enhanced release of Zn^2+^ and Ca^2+^ at pH 4.5–5.5	**Advantages:** Multi-ion synergistic antibacterial effect; responsive release; promotes remineralization. **Disadvantages:** F^−^ release inhibited in acidic environments, reducing anti-caries efficacy	(Liu et al., 2020)
	Eu@B-UiO-66/Zn	MOF composite	Releases eugenol, generates reactive oxygen species, disrupts biofilm	Maintains physical stability for at least 60 days with sustained release potential	**Advantages:** Synergistic pH-responsive release and ROS-based antibacterial action. **Disadvantages:** Potential toxicity from material degradation	(Wang et al., 2024)
	PMs@NaF-SAP	Polymeric micelles	Releases tannic acid and sodium fluoride	Releases 70% TA and 80% NaF within 24 hours at pH 5.0	**Advantages:** Multi-stimuli responsive release; validated both *in vitro* and *in vivo*. **Disadvantages:** Limited drug loading capacity; complex preparation.	(Xu et al., 2023)
Magnetic-Responsive	SPIONs (Fe_3_O_4_)	Magnetic nanocomposite	Release under magnetic field stimulation	Release exceeds 1 mg/mL within 24 hours, sufficient to inhibit MMP-9 activity	**Advantages**: Enhanced bonding strength; combines antibacterial and magnetic-guided penetration. **Disadvantages:** Slightly complex for clinical application.	(Mokeem et al., 2024)
Light/Heat-Responsive	CG-AgPB hydrogel	Hydrogel	NIR-triggered photothermal response, release of Fe^2+^ and Ag^+^, antibacterial and anti-biofilm	808 nm laser irradiation triggers temperature rise >50 °C within 3 minutes	**Advantages:** Synergistic photothermal and ion release for highly effective antibacterial action. **Disadvantages:** Dependent on laser activation.	(Li et al., 2024)
	Sr-ZnO@PDA	Composite material	Yellow light and ultrasound synergistically catalyze ROS generation; Sr^2+^ promotes remineralization	Light/sonication-triggered release of Sr^2+^	**Advantages:** Responsive to multiple physical stimuli; combines antibacterial and remineralization functions. **Disadvantages:** Relatively complex activation method.	(Mu et al., 2025)
	ZnPcS_4_@GC5AF_5_	Supramolecular nanomaterial	660 nm laser triggers PDT/PTT switching mechanism	Light-triggered, ATP environment-adaptive	**Advantages:** Smart switching of treatment mode; high targeting ability; low cytotoxicity. **Disadvantages:** ATP concentration-dependent; complex design.	(Zhang et al., 2024)
Fluoride-rechargeable	CaF_2_ nanoparticles	Resin composite additive	Sustained release of F^−^ and Ca^2+^; PMGDM in resin chelates F^−^ for ion recharge	Enhanced F^−^ release at pH 4.0, rechargeable multiple times	**Advantages:** Long-term fluoride release; rechargeability. **Disadvantages:** HEMA may cause hydrolysis; stringent recharge conditions.	(Yi et al., 2019)
NACP-based	NACP + DMAHDM	Resin composite	NACP releases calcium and phosphate ions under acidic conditions	Low pH triggers NACP release; mechanically triggered microcapsule release	**Advantages**: Self-healing, antibacterial, and remineralizing. **Disadvantages**: High microcapsule content may compromise mechanical properties.	(Wu et al., 2015)
GOx-based	HA@MRuO_2_-Cip/GOx	Nanoreactor	GOx catalyzes glucose to produce acid and H_2_O_2_; pH-triggered antibiotic release and ROS generation	Sustained release of antibiotics and ROS under acidic conditions	**Advantages:** Cascade reaction amplifies antibacterial effect; bacteria-responsive controlled release. **Disadvantages:** High ROS concentrations may damage adjacent normal cells.	(Zhu et al., 2023)
	Na_2_S_2_O_8_@ZIF-67/GOx	ROS nanogenerator	GOx-catalyzed acid production accelerates ZIF-67 decomposition, releasing SO_4_²^−^ and ·OH	At pH 6.5, ZIF-67 decomposes rapidly and the release of ROS can last for 90 minutes	**Advantages:** Highly effective antibacterial; self-acidifying system; multi-mechanism synergy. **Disadvantages:** Complex preparation; potential toxicity risks.	(Ge et al., 2023)
	CoPt@G@GOx	Nanocomposite	Magnetic targeting + two-step cascade reaction to produce ·OH	Continuous production of oxTMB within 6 minutes	**Advantages:** Highly efficient cascade catalysis; magnetic targeting; pH-responsive. **Disadvantages:** Complex preparation.	(Dong et al., 2022)
	MX/AgP-GOx	Heterojunction nanomaterial	NIR photothermal release of Ag^+^ + GOx consumes sugar, synergistic antibacterial effect	Instant response to NIR; release stops upon irradiation cessation; sustained release	**Advantages:** Multi-modal synergy: phototherapy + chemotherapy + metabolic intervention. **Disadvantages**: Risk of accumulation from long-term degradation products of MXene.	(Zhu et al., 2025)
Iron Oxide-based	CAT-NPs	Catalytic nanoparticles	Catalyzes H_2_O_2_ to produce ·OH under acidic conditions, disrupting biofilm	CAT-NP exhibits strongest catalytic activity in acidic environments; capable of killing bacteria and degrading EPS within 5 minutes	**Advantages:** Highly effective antibacterial and anti-biofilm activity; matrix degradation capability; pH-responsive. **Disadvantages:** Potential iron ion accumulation; dependent on H_2_O_2_.	(Gao et al., 2016)
	Ferumoxytol	Nanoparticles	Catalyzes H_2_O_2_ to generate free radicals under acidic conditions, disrupting biofilm	Catalytic reaction initiates within minutes when combined with H_2_O_2_	**Advantages:** High catalytic efficiency; simultaneously kills bacteria and degrades EPS; targets acidic environments. **Disadvantages:** Dependent on exogenous H_2_O_2_.	(Liu et al., 2018a)
	Dex-NZMs	Composite nanoparticles	Dex-NZM catalyzes H_2_O_2_ decomposition into free radicals under acidic conditions	pH-responsive catalysis when applied locally to biofilm surface	**Advantages:** High stability; high catalytic efficiency. **Disadvantages:** Dependent on exogenous H_2_O_2_; limited by pH dependency.	(Naha et al., 2019)

## 2 Stimuli-responsive strategy

Incorporating antimicrobial nano-ions into resin can address bacterial infections that cause secondary caries while maintaining the mechanical strength of the restorative material. Traditional antimicrobial agents often compromise the mechanical properties of resins (Ibrahim et al., 2020). In contrast, nano-antimicrobial agents can effectively kill bacteria at lower concentrations with their high specific surface area and extremely small volume (Sowmya et al., 2024). The nano-structure enriches the antimicrobial mechanisms, including contact killing, inducing cellular oxidative stress, and interfering with metabolism (Jowkar et al., 2025), making it less prone to bacterial resistance—a global concern. Initially, nano-antimicrobial agents faced the challenge of the burst effect: the early release of large amounts of antimicrobial agents caused local drug concentrations to rise, leading to significant cellular toxicity and a sharp decline in antimicrobial efficacy later on (de Jesus et al., 2024).

**Figure 1:**
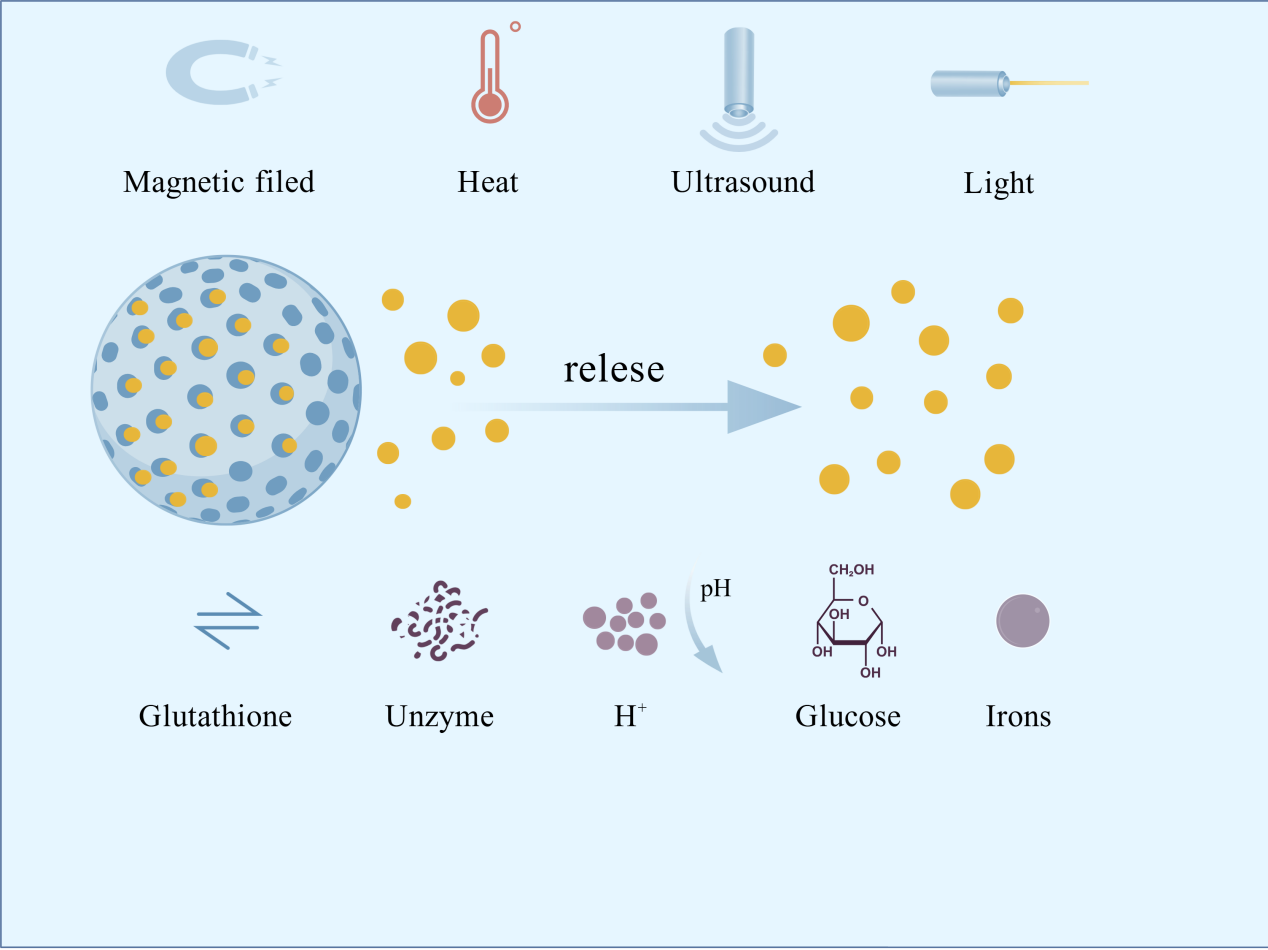
Common stimulus types for nano-responsive materials.

Additionally, concerns arose regarding the accumulation of metal nanoparticles in the body, their potential to induce inflammation, and the risk of breaching the blood-brain barrier (Wang et al., 2023; Yang et al., 2024). Controlled-release nano-antimicrobial materials, which can precisely respond to changes in the oral microenvironment to release antimicrobial agents, have emerged as a highly promising strategy to address this challenge. Common stimulants include exogenous magnetic fields, ultrasound, light, temperature, endogenous glutathione, enzymes, acids, glucose, ions, etc. (**Figure 1**). Light, temperature, and acid stimulants are commonly used to develop oral nanomedicines. Research on pH responsiveness to the special environment created by microbial acid production is the most prevalent.

### 2.1 pH-responsive

Dental caries-causing bacteria, such as *Streptococcus mutans* (*S. mutans*), produce organic acids (lactic acid and acetic acid) through glycolysis. This metabolic process lowers the pH at the interface between the filling material and the tooth structure, ultimately accelerating the dissolution of hydroxyapatite (Sangha et al., 2024).

The mechanism of acid-responsive nanomedicines involves three points: (1) protonation or ionization of functional groups. For example, amino groups (-NH_2_) protonate at low pH values to form -NH_3_^+^, releasing encapsulated antimicrobial agents; (2) cleavage of acid-labile bonds. Aldehyde bonds in specific mesoporous silica nanoparticles readily hydrolyze in acidic environments, enabling rapid drug release; (3) conformational changes in polymers. When pH drops below 5.5, carboxyl groups (–COOH) protonate, causing polymer contraction, which compresses internal pores and expels molecules (Meng et al., 2024; Boyuklieva et al., 2025; Tapponi et al., 2025). Their nanoscale dimensions further enhance biofilm penetration, ensuring efficient drug delivery. When functionalized with fluoride ions (F^-^), these systems exhibit synergistic antimicrobial-remineralizing dual functionality, presenting a promising strategy for secondary caries prevention and therapy (Qi et al., 2025). Currently, acid-stimuli-responsive nanomaterials are the most extensively studied. Below, three typical acid-responsive materials are introduced: BioUnion, Eugenol, and Tannic acid.

#### 2.1.1 BioUnion

BioUnion filler is a novel bioactive glass powder (Xia et al., 2025), that addresses the issue of declining fluoride release over time observed in traditional glass ionomer cements (GICs) (Dcruz et al., 2024; Da et al., 2025). It composed of silicon dioxide (SiO_2_), zinc oxide (ZnO), calcium oxide (CaO), and a fluoride compound. Its particles can be incorporated into dental materials, releasing Zn²^+^ (combats oral bacteria and reduces dentin demineralization), F^−^ (inhibits demineralization), and Ca^2+^ (enhances remineralization) ions to prevent secondary caries (Fallahzadeh et al., 2022; Piszko et al., 2025). It also exhibits pH-dependent selective release of Zn^2+^/Ca^2+^ (Liu et al., 2020).In bovine dentin subjected to 4-week pH-cycling, the GICs-Bio group (Control group: GICs) demonstrated superior inhibition of demineralization and promotion of remineralization. *In vitro* salivary titration assembly revealed that BioUnion™-incorporated GIC provides higher initial fluoride release and a more sustained release profile under simulated oral conditions (Htet et al., 2025).

#### 2.1.2 Eugenol

Whereas BioUnio’s strategy relies on antibacterial action and remineralization, eugenol acts primarily through pathogen inhibition and pulp inflammation modulation to prevent secondary caries (Nazemisalman et al., 2024; Patil et al., 2025). Innovative nano-delivery systems now enhance its efficacy by improving solubility and enabling controlled release.

These systems retain Eugenol’s functionality while achieving slow, sustained drug release, and can be triggered to release in response to specific environmental stimuli, thereby avoiding the cytotoxicity associated with long-term high-concentration release, improving biosafety (Khan et al., 2019; Montoya et al., 2023). Specific applications include the Eu@B-UiO-66/Zn system, based on eugenol-loaded B-UiO-66 MOF complexed with Zn^2+^, which achieves pH-responsive release via a phenolic hydroxyl-Zn^2+^ cage-like network where the released Eugenol generates reactive oxygen species (ROS) to disrupt biofilms (Wang et al., 2024); nanoencapsulation significantly enhances eugenol stability, for example up to 60 days (Vilela et al., 2023);acid-triggered nanoparticles modified with casein and based on hydroxyapatite/calcium carbonate that adhere to dentin and release Eugenol under low pH conditions (Sereda et al., 2023); synthetic eugenol derivatives containing polymerizable methacrylate groups, such as EgMA, enabling participation in resin free-radical polymerization, which maintain antibacterial activity against Gram-positive(G^+^) and Gram-negative(G^-^) bacteria while avoiding detrimental effects on resin polymerization and strength (Almaroof et al., 2016).

#### 2.1.3 Tannic acid

Nanotechnology not only overcomes the limitations of natural active substances but also endows them with new functionalities. Tannic acid (TA) is a water-soluble natural polyphenol extracted from plants with antibacterial properties (Yamauchi et al., 1989; Sileika et al., 2013). When combined with phenylboronic acid (PBA), it forms a borate ester bond that can break down and release functional molecules under pH changes caused by bacteria (Springsteen and Wang, 2002). Using nano-polymer micelles (PM) with a shell-core structure as a scaffold, Sodium fluoride (NaF), tannic acid, and salivary acquired peptides (SAP)were loaded to produce multifunctional smart-release nano-antimicrobial materials PMs@NaF-SAP. At pH 5.0, nearly 70% of TA was released from PMs@NaF -SAP, while only approximately 40% was released at pH 7.4. Additionally, the more acidic the pH, the faster the NaF release rate; at pH 5.0, approximately 80% of NaF is released within 24 hours, and its antibacterial efficacy at this pH value is significantly stronger than that of the chlorhexidine (CHX) control group. As determined, PMs@NaF-SAP exhibits excellent demineralization inhibition and remineralization promotion capabilities, with relatively smooth tooth surfaces. Results from a caries-prone rodent model indicate that PMs@NaF-SAP treatment reduces the incidence and severity of lesions on smooth and fissure surfaces, effectively preventing the development of caries *in vivo* (Xu et al., 2023).

### 2.2 Magnetic field-responsive

Magnetically responsive materials, activated by an external magnetic field, enable the stimulation or targeting of specific objects using materials containing magnetic substances (Menezes-Silva et al., 2021). The magnetic field can adjust ion distribution within the material, enhancing the marginal sealing of restorative materials and reducing microleakage. Under its guidance, nanoparticles can penetrate dentinal tubules effectively, improving bonding strength.

Superparamagnetic iron oxide nanoparticles (SPIONs) exhibit non-magnetic behavior without an external magnetic field. However, when subjected to an external field, they respond rapidly to Lorentz forces, enabling their movement or directional alignment under magnetic guidance (Hu et al., 2017). In dental adhesives, SPIONs (Fe_3_O_4_) guided by a magnetic field can infiltrate the micropores of dentin more effectively, leading to enhanced bond strength. This process generates mild thermal effects that can inhibit bacterial growth (Garcia et al., 2021). Ferroferric Oxide (Fe_3_O_4_) is a type of SPION. Constructing a core-shell structure with Fe_3_O_4_ cores and mesoporous silica shells allows for encapsulating antimicrobial agents like CHX and quaternary ammonium silane. This system achieved an inhibition rate of over 78% against *S. mutans* biofilms and maintained antibacterial activity even after 6 months of artificial aging, with no significant cytotoxicity observed (Mokeem et al., 2024). Targeted, sustained antimicrobial delivery to the critical interface prolongs restoration longevity.

Magnetic field-responsive materials provide superior spatiotemporal control and deep-tissue activation capabilities but at the cost of requiring external hardware. Acid-responsive materials are simpler, more autonomous, and highly biocompatible, but their action is less precise and confined to acidic micro-environments.

### 2.3 Light/heat responsive

For clinical ease-of-use, photothermal-responsive systems are ideal, as they allow therapeutic agents to be activated on-demand with a straightforward light source. Traditional nanomaterials exert antibacterial effects by releasing metal ions such as Ag^+^, Cu^2+^, and Zn^2+^, which disrupt bacterial cell membranes and interfere with enzymatic activities (Xiu et al., 2011; Fan et al., 2021). However, their “burst release” mechanism leads to short therapeutic duration and lacks precise control (Jiang et al., 2023). Photo-responsive nanomaterials based on photodynamic therapy (PDT) offer a novel approach for preventing and managing secondary caries. PDT involves three key components: oxygen, an excitation light source, and a photosensitizer (PS). When exposed to light of specific wavelengths, the PS generates toxic ROS. These ROS possess strong oxidizing power and high reactivity, thereby inducing rapid lipid peroxidation in bacteria (Pereira et al., 2013; Fiegler-Rudol et al., 2025).

Photothermal therapy (PTT) and PDT are two complementary and promising phototherapeutic approaches. Silver-ion-doped Prussian Blue (AgPB) nanoparticles were encapsulated in cationic guar gum (CG) to form an antibacterial PTT hydrogel, CG-AgPB, exhibiting a photothermal conversion efficiency of 34.4%. Upon exposure to an 808 nm laser at a power density of 0.4 W cm², the hydrogel surpassed 50 °C within 3 minutes, synchronized with the release of Ag^+^ ions from interstitial sites of the AgPB lattice, thereby inhibiting both individual oral cariogenic bacteria and their biofilms. *In vivo*, CG-AgPB-mediated PTT significantly reduced cariogenic bacteria to less than 1% of the original load in a rat caries model (Li et al., 2024). The laser power and irradiation duration can be precisely modulated to confine the therapeutic thermal zone, thereby minimizing collateral damage to adjacent healthy tissues (Alumutairi et al., 2020).

A polydopamine (PDA)-coated strontium-doped zinc oxide composite (Sr-ZnO@PDA) responds to yellow light and ultrasound. Synergistic piezophotocatalysis generates ROS, destroying bacterial cell membrane structures and decomposing dental surface pigments. Additionally, strontium ions (Sr^2+^) released from SZ@PDA promote remineralization of enamel and dentin, repairing damaged tooth tissues (Mu et al., 2025). Another supramolecular nanoformulation achieves highly efficient inhibition of *S. mutans* biofilms through an adaptive PDT/PTT switching mechanism. Guanidinium groups on the material surface specifically bind to negatively charged moieties such as lipoteichoic acid and ATP on the *S. mutans* cell membrane, enabling targeted accumulation. Upon 660 nm laser irradiation, synergistic PTT and PDT effects are observed. In low-ATP environments (planktonic bacteria), the photosensitizer ZnPcS_4_ exists as monomers that predominantly generate ROS via PDT, disrupting bacterial membrane integrity. In high-ATP environments (biofilms), ZnPcS_4_ forms H-aggregates that chiefly produce heat through PTT for bactericidal action, circumventing the limited ROS penetration issue. Experiments also confirmed the material’s low cytotoxicity; compared to the markedly demineralized control group, enamel remained notably intact in a rat dental caries model (Zhang Y. et al., 2024).

This section highlights the promise of stimuli-responsive nanomedicines for combating secondary caries. pH-responsive systems offer high clinical relevance by leveraging the cariogenic microenvironment to trigger targeted drug release and demonstrate synergistic antibacterial-remineralization effects. In comparison, external field-responsive strategies provide superior spatiotemporal control over treatment, enabling on-demand activation. Yet they face significant practical limitations. For light-responsive systems in particular, limited tissue penetration depth remains a major constraint. In morphologically complex teeth such as molars, light may be unable to effectively reach lesions in proximal or deep dentinal areas, resulting in incomplete treatment. Similarly, magnetic-responsive approaches require externally applied devices, which may complicate clinical integration and routine use.

## 3 Rechargeable strategy

**Figure 2:**
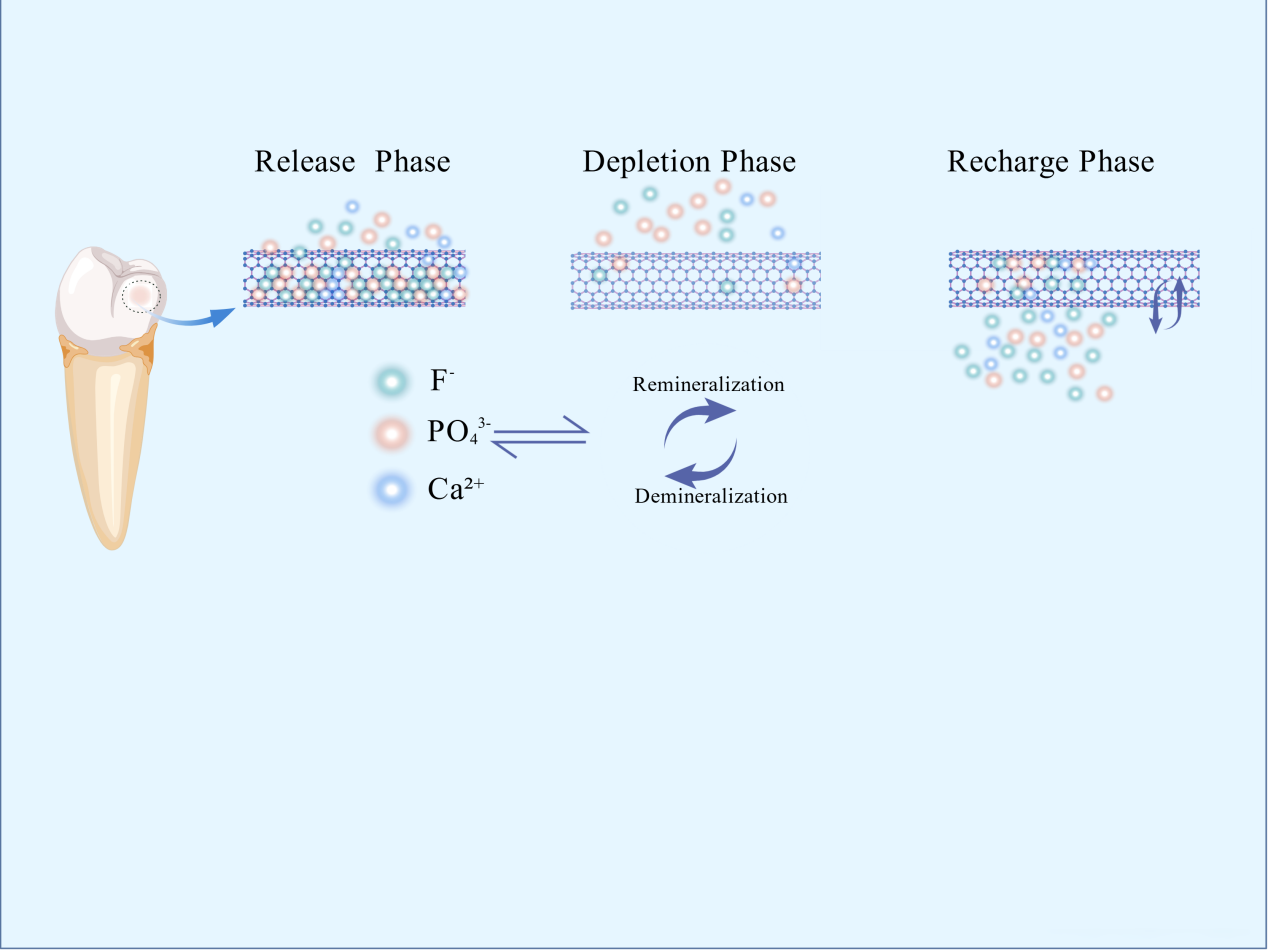
Mechanism of rechargeable nanomaterials.

Interventions for secondary caries primarily encompass antibacterial approaches and remineralization. For years, researchers have incorporated remineralizing ions into restorative materials. Fluoride ions(F^-^) facilitate the deposition of calcium ions (Ca^2+^) and phosphorus ions (PO_4_³^−^) within the tooth structure, forming more acid-resistant fluorapatite (Ca_5_(PO_4_) _3_F), while also buffering the acidic environment created by bacteria. Sodium fluoride nanoparticles have reduced secondary caries at restoration margins by releasing F^-^ and Ca^2+^ (Albelasy et al., 2023). However, the short duration of effective ion release from these materials, relative to the expected lifespan of restorations, remains a critical challenge. To address this, a rechargeable strategy has been proposed to prolong ion release (Kelić et al., 2023). This approach centers on replenishing the diminishing active ions within the restorative material through exogenous ion exchange, restoring its remineralizing potential. There are three phases: 1) Release phase: Soluble active ions (F^−^/Ca^2+^) are released from the material, promoting remineralization; 2) Depletion phase: Continued ion release depletes surface reservoirs, leading to declining ion concentration and diminished remineralization capacity; 3) Recharging phase: The material is exposed to an exogenous high-concentration ion solution. Driven by concentration gradients or ion exchange, new ions diffuse into the inorganic matrix/microporous structure, achieving reloading (Puttipanampai et al., 2025) (**Figure 2**).

### 3.1 Fluoride

As widely documented, fluoride combats dental caries through three mechanisms: inhibiting demineralization, promoting remineralization, and suppressing bacterial metabolism (Monjarás-Ávila et al., 2025). To address the transient fluoride release from existing restorative materials, rechargeable fluoride nanomaterials offer a new way for managing secondary caries.

Calcium fluoride (CaF_2_) nanoparticles (average 58 nm), synthesized via spray-drying and incorporated into resin matrices, exhibit excellent biocompatibility and significantly enhanced F^−^ release capacity that remains stable over repeated recharging cycles (Yi et al., 2019). These materials demonstrate high pH sensitivity, where acidic conditions trigger a surge in fluoride release, particularly pronounced in systems containing CaF_2_ or fluorohydroxyapatite under cariogenic pH (4.5-5.5) generated by bacterial metabolism (Morawska-Wilk et al., 2025). Studies confirm that ion release (Ca^2+^, F^−^, PO_4_³^−^) at acidic pH (4.5-5.5) is markedly higher than at neutral pH (6.5), a trend sustained even after recharging (Venkataiah et al., 2025). This pH-responsive sustained release is critical in resin-based restorations: it enhances remineralization within cariogenic microenvironments and extends restoration longevity. Both silane- and methacrylate-based composites effectively serve as rechargeable fluoride carriers (Madhyastha et al., 2025). The synergy between acid-triggered release and on-demand recharging holds significant promise for combating secondary caries.

### 3.2 Nanoamorphous calcium phosphate

Amorphous calcium phosphate (ACP) possesses a non-crystalline structure and exhibits high ionic release activity (Degli Esposti and Iafisco, 2022). Nanotechnology has effectively addressed the challenge of uncontrolled ion release from traditional ACP. Under acidic conditions, NACP rapidly releases significant amounts of calcium (Ca^2+^) and phosphate (PO_4_³^−^) ions, while maintaining structural stability with minimal ion release in neutral or alkaline environments (Liang et al., 2019).

Incorporating nano-sized NACP alongside dimethylaminohexadecyl methacrylate (DMAHDM) nanoparticles into resin-based materials creates a dual-functional composite. The Ca^2+^ and PO_4_³^−^ ions released from NACP neutralize acids produced by cariogenic bacteria, disrupting the acidic environment conducive to demineralization and promoting remineralization. Simultaneously, the DMAHDM component exerts a potent direct antibacterial effect (Wu et al., 2015; Sales-Junior et al., 2025). Furthermore, animal models have demonstrated that these composites induce minimal pulp irritation and stimulate tertiary dentin formation (Li et al., 2014). In an *in vitro* saliva-derived biofilm secondary caries model, it inhibits the growth of cariogenic bacteria at the resin margin and has no negative impact on tooth enamel hardness (Zhou et al., 2020).

NACP has also been successfully integrated into dental adhesives, often with monobasic calcium phosphate dihydrate (MCPM), to enhance their remineralization and antibacterial capabilities (Liu et al., 2018a). Despite finite ion release duration (typically months), rechargeable NACP nanocomposites overcome this limitation via periodic calcium/pHospHate solution immersion. Both rechargeable NACP and NACP-DMAHDM variants retain flexural strength and elastic modulus comparable to commercial counterparts. They significantly inhibit biofilm metabolic activity and lactic acid production, while drastically reducing biofilm colony-forming units (CFUs). A single recharge sustains effective ion release for up to 42 days (Al-Dulaijan et al., 2018). Crucially, the release profile remains stable over multiple recharge cycles, demonstrating long-term ion release and remineralization potential. This recharge strategy also shows significant anti-caries efficacy in adhesives and sealants (Ibrahim et al., 2020). For instance, a novel self-healing dental adhesive incorporating poly(urea-formaldehyde) (PUF) microcapsules, DMAHDM, and NACP achieved a 67% crack-healing efficiency without compromising dentin bond strength. Concurrently, it reduced biofilm CFUs by four orders of magnitude, confirming its potent antibacterial properties (Wu et al., 2019).

This rechargeable strategy substantially bridges the translational gap: Recharging requires mere minutes per session, with a single treatment sustaining caries-preventive efficacy for up to six months. Crucially, at-home recharge via mouth rinse empowers patient autonomy. It establishes a clinically viable protocol for long-term dynamic management of secondary caries.

Rechargeable resin-based restoratives can markedly extend the functional lifespan of dental restorations through their replenishable ion-release mechanism, simultaneously enhancing remineralization and antimicrobial efficacy. In acidic environments, these materials exhibit intelligent, pH-responsive ionic discharge while maintaining favorable biocompatibility and mechanical stability, offering a novel long-term dynamic management strategy for recurrent caries.

Nevertheless, recent investigations have highlighted several shortcomings. First, although CaF_2_ and NACP nanoparticles are intrinsically cytocompatible, high fluoride loads or DMAHDM quaternary ammonium antimicrobials still display concentration-dependent cytotoxicity in 3-D spheroid cultures or human dental-pulp stem-cell assays, compromising the biosafety of the pulp and surrounding soft tissues (Fei et al., 2024). Second, even advanced CAD/CAM resin composites undergo inevitable deterioration in flexural strength and surface hardness after prolonged exposure to the moist oral milieu, primarily attributable to water sorption and hydrolytic degradation (Wendler et al., 2021). Third, fluoride-releasing materials experience significant deterioration of nanohardness, elastic modulus, and surface roughness when challenged by acidic beverages (Güner and Köse, 2024). Fourth, no consensus exists on the concentration, pH, or contact time of “recharge mouthwashes”; regulatory agencies have yet to establish *in vitro*–*in vivo* correlation guidelines, and published clinical trials are underpowered with follow-up periods < 2 years, precluding reliable assessment of long-term marginal integrity and secondary-caries incidence (Zhang J. et al., 2024).

## 4 Cascade catalytic nanoreactor

**Figure 3:**
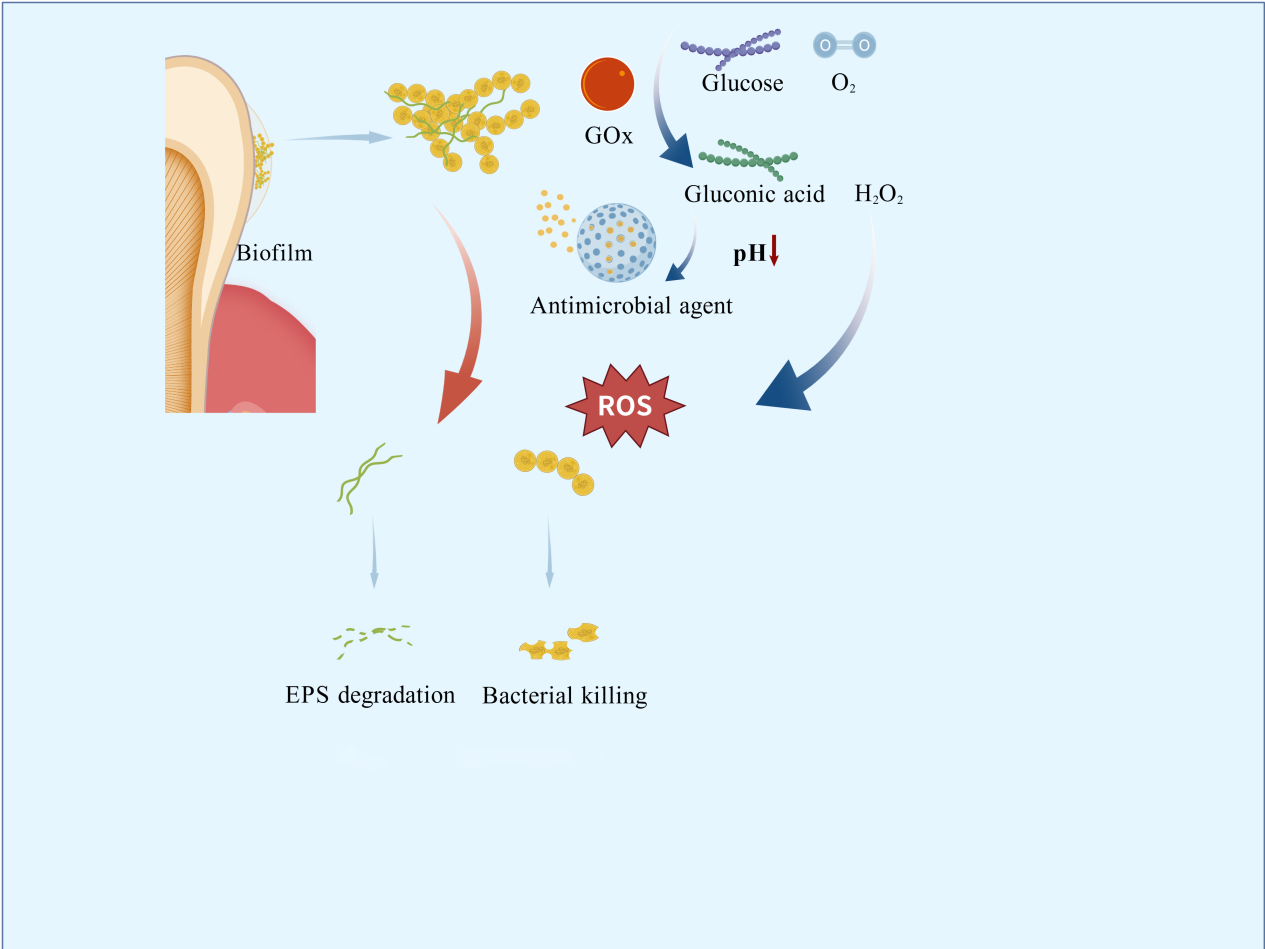
Mechanism of antimicrobial cascade nanoreactors.

Integrating nanotechnology, catalytic chemistry, and biomedicine has led to the development of cascade catalytic nanoreactors, representing an advanced strategy for preventing and treating oral infectious diseases. Inspired by intracellular multi-enzyme cascade reactions, researchers initially attempted to construct artificial cascade systems by encapsulating natural enzymes within porous synthetic materials, including metal-organic frameworks (MOFs), covalent organic frameworks (COFs), and hydrogen-bonded organic frameworks (HOFs) (Lykourinou et al., 2011; Huang et al., 2022). However, these early systems suffered from poor enzyme stability, low reaction efficiency, and inadequate controllability (Meghwanshi et al., 2020). Subsequent advancements replaced natural enzymes with inorganic nanocatalysts (Fe_3_O_4_ or Au nanoparticles), effectively resolving stability issues (Li et al., 2015; Kwon et al., 2021; Ma et al., 2024). Further innovation incorporated stimuli-responsive materials (ultrasound or pH-activated components) to achieve precisely controlled activation (Chen and Li, 2020; Li et al., 2022). Self-supplying substrate systems were engineered to overcome dependence on external reactants (**Figure 3**). Consequently, application scenarios have expanded beyond tumor therapy to encompass anti-biofilm interventions.

### 4.1 Glucose oxidase

GOx exhibits catalytic properties and bioactivities uniquely suited to the cariogenic microenvironment: it oxidizes glucose and oxygen into gluconic acid and hydrogen peroxide (Dubey et al., 2017). This reaction directly depletes free glucose, competing with *S. mutans* for metabolic substrates and suppressing bacterial growth and acid production (Senol et al., 2007; Yuan et al., 2015). The in situ-generated hydrogen peroxide reduces glucosyltransferase activity, diminishing viscous glucan synthesis and disrupting biofilm formation mechanisms (Zhang et al., 2021). Concurrently, gluconic acid production triggers pH-responsive drug release (Wang et al., 2022). GOx is not used in isolation. It is integrated into nanocomposites that incorporate additional targeting strategies (such as magnetic guidance, pH-responsive release, or photothermal activation) which enhance spatial precision and biological specificity. GOx does not target specific bacterial species through molecular recognition, but rather functionally discriminates against cariogenic pathogens. These targeted mechanisms establish a novel paradigm for constructing multimodal antimicrobial systems.

The greatest advantage of cascade nanoreactors lies in their ability to integrate and leverage distinct molecular functions. The HA@MRuO_2_–Cip/GOx composite (composed of hydroxyapatite, HA; mesoporous ruthenium dioxide, MRuO_2_; ciprofloxacin, Cip; and GOx) incorporates a mesoporous RuO_2_ core co-loaded with Cip and GOx. *In vitro*, the composite disrupts biofilms by cleaving extracellular DNA, thereby enhancing the bactericidal efficacy of Cip against planktonic bacteria. Under acidic conditions, it not only sustains ROS generation but also allows controlled release of antibiotics (Zhu et al., 2023).

Similarly leveraging acidic conditions for antibacterial activity, Na_2_S_2_O_8_@ZIF-67/GOx(composed of sodium persulfate, Na_2_S_2_O_8_; zeolitic imidazolate framework-67, ZIF-67; and GOx) functions as a novel ROS nanogenerator. GOx-mediated catalysis in acidic microenvironments produces H_2_O_2_ and gluconic acid, which further reduces pH to accelerate ZIF-67 decomposition and Na_2_S_2_O_8_ release. This process yields highly toxic sulfate (SO_4_^2−^) and hydroxyl (-OH) radicals, effectively suppressing bacterial proliferation and biofilm formation (Ge et al., 2023).

Also operating under external energy stimulation, the nanozyme CoPt@G@GOx (composed of cobalt-platinum alloy, CoPt; graphene, G; and GOx) employs a two-step cascade reaction to generate potent -OH radicals in acidic environments, achieving 4-fold enhanced catalytic efficiency in disrupting *S. mutans* biofilms compared to simple mixtures. Its magnetic CoPt@grapHene core enables precise targeting of carious lesions via external magnetic fields, minimizing off-target effects (Dong et al., 2022). TiO_2_-GOx(composed of titanium dioxide, TiO_2_; and GOx)exhibits high catalytic activity in glucose-rich environments under UV irradiation, significantly amplifying ROS production (Kim et al., 2019).

Incorporating both photothermal and biochemical antibacterial mechanisms, MX/AgP-Gox (composed of MXene, MX; silver nanoparticles, AgNPs; and GOx) is a cascade bio-heterojunction (HJ) engineered to target both dental-plaque biofilms’ chemical and biological constituents. Under near-infrared (NIR) irradiation, the HJ rapidly heats up to generate photothermal effects while exploiting H_2_O_2_ to burst-produce massive ROS, achieving highly efficient phototherapy. Even in darkness, the bactericidal action of Ag^+^ ions and glucose depletion mediated by GOx act synergistically to suppress bacteria, ensuring long-lasting anti-caries efficacy after light withdrawal. Rat caries models further confirmed that the material inhibits enamel demineralization and exhibits excellent biocompatibility (Zhu et al., 2025).

### 4.2 Iron oxide

Catalytic iron oxide nanoparticles (CAT-NPs) mimic natural peroxidases through their intrinsic catalytic activity. Under acidic conditions, they catalyze H_2_O_2_ decomposition into hydroxyl radicals (-OH), disrupting bacterial membranes, degrading intracellular macromolecules, and breaking down extracellular polymeric substances (EPS) within biofilms. *In vitro* studies demonstrate that CAT-NPs reduce apatite demineralization at acidic pH. Topical application combined with H_2_O_2_ effectively suppresses caries initiation and progression without adverse effects on oral mucosal tissues *in vivo* (Gao et al., 2016).

Ferumoxytol is a Food and Drug Administration (FDA)-approved nanoparticle formulation used for magnetic resonance imaging and the treatment of iron deficiency (Dósa et al., 2011). Ferumoxytol can penetrate the interior of biofilms and, under acidic conditions, catalyze the production of reactive radicals from H_2_O_2_. These radicals disrupt bacterial cell membranes and degrade extracellular polysaccharide matrices, killing the bacteria. In ex vivo biofilm models, its combination with low concentrations of H_2_O_2_ effectively inhibits biofilm accumulation on natural teeth and prevents enamel demineralization (Liu et al., 2018b). Notably, it enables pathogenic biofilm detection via colorimetric responses, establishing a theranostic platform for caries management (Liu et al., 2021). Dextran-coated iron oxide nanoparticles (Dex-NZMs) maintain catalytic efficiency while enhancing stability. These particles exhibit potent catalytic activity at acidic pH, enabling biofilm-specific targeting. Dex-NZMs prevent severe carious lesions in murine caries models while demonstrating excellent biosafety (Naha et al., 2019).

Cascade-catalytic nanoreactors that emulate natural enzymatic cascades have recently emerged as promising therapeutics for oral infectious diseases. They efficiently generate ROS, respond precisely to the pathological microenvironment, supply their own substrates, and exert multimodal synergistic antimicrobial effects. Collectively, these attributes substantially enhance biofilm eradication while minimizing collateral damage to healthy tissues.

Nevertheless, clinical translation remains hindered by several unresolved limitations. Catalytic activity is prone to attenuation in the complex oral milieu owing to protein adsorption, pH fluctuations, and variable ionic strength, leading to suboptimal durability and limited functional persistence (Ming et al., 2020). Moreover, the fidelity of cascade activation is still imperfect; off-target triggering or adventitious side-reactions may compromise therapeutic predictability and patient safety (Zhang et al., 2018). It constitute an innovative therapeutic platform, yet scalable manufacturing, reproducible quality control, and comprehensive regulatory frameworks are still in their infancy.

## 5 Opportunities and challenges in clinical translation

The heterogeneity of biofilms and the presence of salivary proteins pose challenges for oral antibacterial materials. The former has long posed a significant challenge to antimicrobial materials: the oral biofilm forms a complex three-dimensional structure. The EPS matrix acts like a “fortress wall” that blocks the penetration of antimicrobial molecules (Zhao et al., 2023). Gradients of oxygen and nutrients from the surface to the deeper layers lead to altered bacterial metabolic activity, with dormant bacteria in the deep layers exhibiting stronger drug tolerance (Liu et al., 2024).

Controlled-release nano-antimicrobial materials show promising performance in addressing biofilm heterogeneity. Physically, their nanoscale structure provides high surface energy, enhancing adhesion to negatively charged bacterial biofilms and enabling penetration through the EPS matrix to deliver antimicrobial agents directly to dormant bacteria in the deeper layers (Han et al., 2022). Moreover, the released antimicrobial molecules can disrupt bacterial membrane potential, inhibit enzymatic activity, and promote ROS generation (Sahli et al., 2022). Finally, through controlled-release mechanisms, these materials maintain effective bactericidal concentrations at the infection site, preventing exposure to sublethal doses and thereby suppressing the enrichment of drug-resistant mutants. These advantages make them highly effective against biofilms.

However, salivary proteins present a double-edged sword. Within hours after cleaning, teeth become coated with a layer of salivary proteins, which can isolate antibacterial materials from bacteria, thereby reducing antimicrobial efficiency and long-term efficacy (Heo et al., 2013). At the same time, this protein layer facilitates bacterial adhesion. However, current nano-antibacterial materials often fail to account for this effect. How to resist salivary protein adhesion through surface modification of nanomaterials is thus an important issue. Research should also be conducted under conditions that simulate the oral environment to more accurately evaluate the practical antibacterial performance of these materials. Rather than focusing solely on avoiding salivary proteins, it would be more beneficial to explore how to leverage them to enhance material functionality.

**Figure 4:**
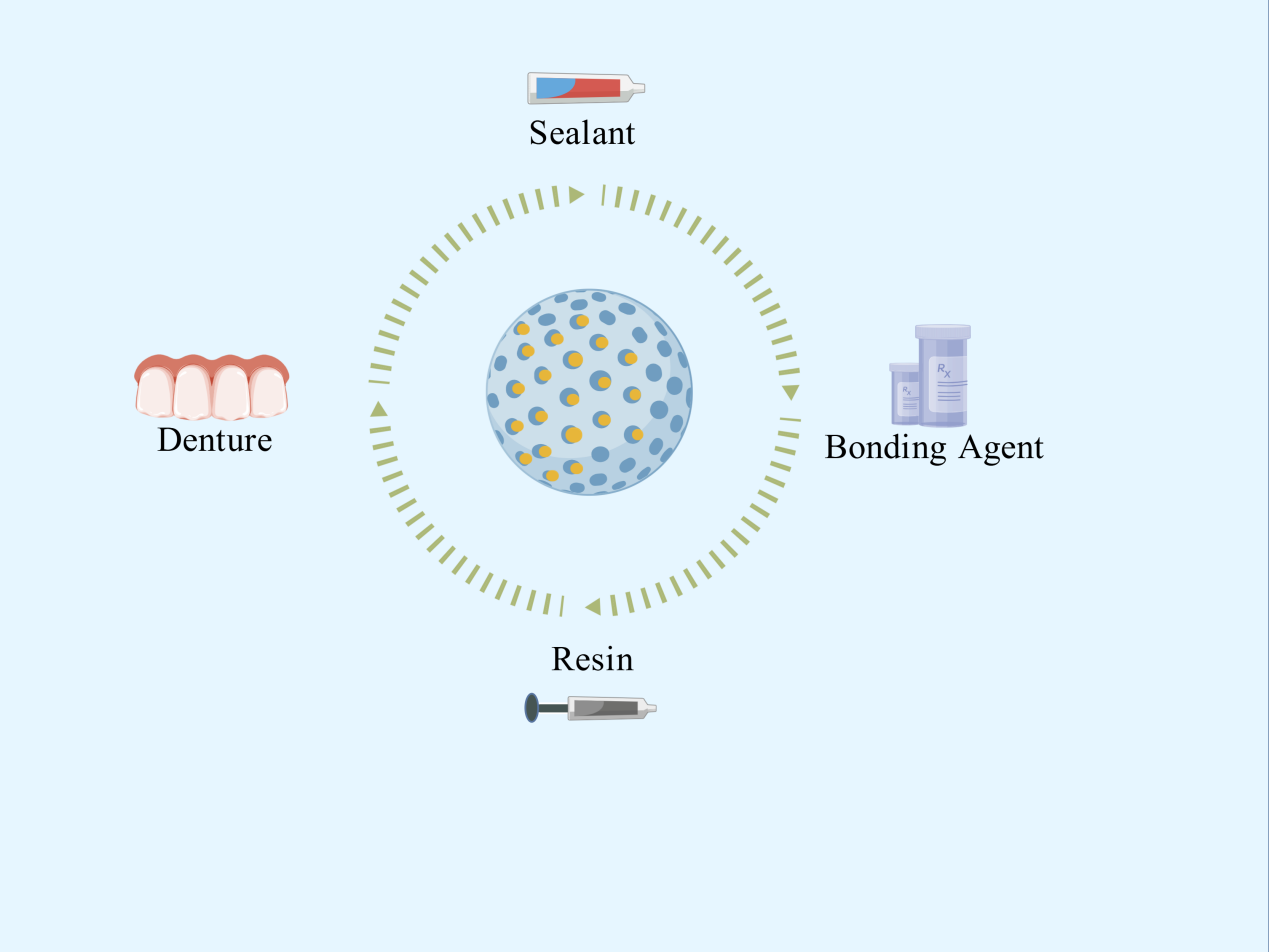
Applications of controlled-release nano-antimicrobial materials.

In addition to the two aforementioned oral-specific environmental resistance factors, the clinical translation of nanomaterials currently faces several challenges:(1) the long-term biosafety, biocompatibility, and functional durability of these nanomaterials within the oral cavity require rigorous assessment in live animal models and, ultimately, clinical trials (Sajithkumar et al., 2025). Many studies discussed in this article are primarily *in vitro*. Not only in the dental field, one of the biggest current challenges for nanomaterials is the lack of long-term *in vivo* experiments. While a few studies have employed rodent caries models to show efficacy in reducing lesion severity, these are often short-term and lack evaluation of critical endpoints; (2) the regulatory landscape for nano-enabled medical products remains fragmented. Harmonized characterization standards and quality-control guidelines are urgently required. Critical parameters—including particle-size distribution, surface charge, and degradation products—must be quantified through validated, standardized assays to guarantee batch-to-batch consistency and clinical reproducibility (Rodríguez-Gómez et al., 2025). (3) Since some dental materials need to meet aesthetic requirements, the coloration issue of certain metal nanoparticles also cannot be ignored. Despite these challenges, the unique properties of controlled-release nano-antimicrobial materials have led to their exploration in a wide range of dental applications. These applications aim to leverage their sustained and targeted antibacterial action to improve the longevity and success of various dental treatments and restorative procedures (**Figure 4**).

## 6 Conclusion

This paper systematically reviews the latest mechanistic advances in controlled-release nanomaterials for preventing secondary caries. Stimulus-responsive systems have evolved from single pH triggers to multi-stimulus synergistic activation, rechargeable nanocarriers have graduated from one-off ion reservoirs to “stimulus–response–re-supply” cycles, and cascade nanoplates now seamlessly integrate antibacterial, acid-neutralizing, and remineralizing functions for precise modulation of the cariogenic microenvironment.

However, the oral pH landscape is constantly perturbed by diet, saliva and microbial metabolism, making it difficult for materials to respond specifically to acid niches generated solely by cariogenic bacteria; The layer-by-layer fabrication of cascade structures still relies on complex processes, which limits large-scale production and cost control; and unified characterization protocols together with long-term biosafety regulation for nanomaterials are still absent. These challenges are compounded by patient compliance issues, as complex or expensive preventive measures may lead to poor adherence, reducing real-world effectiveness. Therefore, overcoming these technical and economic barriers while ensuring user-friendly and affordable solutions, which is essential to advancing effective caries management.
